# Prediction of *matrilineal* specific patatin-like protein governing *in-vivo* maternal haploid induction in maize using support vector machine and di-peptide composition

**DOI:** 10.1007/s00726-023-03368-0

**Published:** 2024-03-09

**Authors:** Suman Dutta, Rajkumar U. Zunjare, Anirban Sil, Dwijesh Chandra Mishra, Alka Arora, Nisrita Gain, Gulab Chand, Rashmi Chhabra, Vignesh Muthusamy, Firoz Hossain

**Affiliations:** 1 ICAR-Indian Agricultural Research Institute, New Delhi, India; 2 ICAR-Indian Agricultural Statistical Research Institute, New Delhi, India

**Keywords:** Haploid induction Patatin-like phospholipase, SVM, Kernels, Feature selection

## Abstract

The mutant *matrilineal* (*mtl*) gene encoding patatin-like phospholipase activity is involved in *in-vivo* maternal haploid induction in maize. Doubling of chromosomes in haploids by colchicine treatment leads to complete fixation of inbreds in just one generation compared to 6–7 generations of selfing. Thus, knowledge of patatin-like proteins in other crops assumes great significance for *in-vivo* haploid induction. So far, no online tool is available that can classify unknown proteins into patatin-like proteins. Here, we aimed to optimize a machine learning-based algorithm to predict the patatin-like phospholipase activity of unknown proteins. Four different kernels [radial basis function (RBF), sigmoid, polynomial, and linear] were used for building support vector machine (SVM) classifiers using six different sequence-based compositional features (AAC, DPC, GDPC, CTDC, CTDT, and GAAC). A total of 1170 protein sequences including both patatin-like (585 sequences) from various monocots, dicots, and microbes; and non-patatin-like proteins (585 sequences) from different subspecies of *Zea mays* were analyzed. RBF and polynomial kernels were quite promising in the prediction of patatin-like proteins. Among six sequence-based compositional features, di-peptide composition attained > 90% prediction accuracies using RBF and polynomial kernels. Using mutual information, most explaining dipeptides that contributed the highest to the prediction process were identified. The knowledge generated in this study can be utilized in other crops prior to the initiation of any experiment. The developed SVM model opened a new paradigm for scientists working in *in-vivo* haploid induction in commercial crops. This is the first report of machine learning of the identification of proteins with patatin-like activity.

**Supplementary Information:**

The online version contains supplementary material available at 10.1007/s00726-023-03368-0.

## Introduction

Production of doubled haploid (DH) in maize has emerged as an integral component in commercial breeding programmes (Gain et al. 2022). Development of inbreds using DH requires 1–2 generations as compared to 6–7 generations using conventional selfing (Dutta et al. 2022). DH lines are created through *in-vivo* and *in-vitro* methods. *In-vitro* is not widely used to achieve the long-term breeding goal due to the involvement of more cumbersome regeneration protocols and well-equipped labs coupled with trained technical personnel. On the contrary, the *in-vivo* method has become an attractive and logistic choice for large production of homozygous lines in maize (Chaikam et al. 2019). Breakthrough came when a naturally existing mutant designated as ‘Stock 6’ was discovered in maize which showed a haploid induction rate (HIR) higher (< 3%) than the normal maize (Coe 1959). Later on, several haploid inducers were developed in different countries based on the ‘Stock 6’ derived mutant line (Prasanna et al. 2012). Improved haploid inducers with 6–15% HIR have been achieved in recent years (Chaikam et al. 2018). Following haploid production, doubled haploid (DH) plants are generated through the doubling of chromosomes using colchicine treatment (Dutta et al. 2022).

The underlying genomic region for *in-vivo* maternal haploid production in maize was designated as *qhir1* QTLs on chromosome 1 (bin 1.04) explaining 66% of the phenotypic variation for haploid induction rate (Dong et al. 2013). Later, the underlying gene, *matrilineal* (*mtl*) (Kelliher et al. 2017) or *Not Like Dad* (*nld*) (Gilles et al. 2017) or *ZmPLA1* (Liu et al. 2017) encoding patatin-like phospholipase was identified as the candidate for haploid induction in maize. Patatin-like protein generally possesses non-specific lipolytic acyl hydrolase (LAH) activity, which catalyzes the hydrolysis of the galactolipids mono galactosyl diacylglycerol (MGDG) and di-galactosyl diacylglycerol (DGDG) (Camera et al. 2005). When one galactose residue is linked to C3 of a di-acylglycerol, it is termed as MGDG, whereas DGDG contains two galactose residues (Kobayashi et al. 2007). Both MGDG and DGDG are important galactolipids for thylakoid membrane biosynthesis as the final step of MGDG pathway occurs in a plastid envelope catalyzed by MGDG synthase enzyme (Kobayashi et al. 2007). Patatin-like protein catalyzes less efficiently for the hydrolysis of phospholipids namely phosphatidylcholine (PC), phosphatidylethanolamine (PE), phosphatidylglycerol (PG), phosphatidic acid (PA), phosphatidylserine (PS) and phosphatidylinositol (PI) (Camera et al. 2005). In *Arabidopsis,* one patatin-like phospholipase was found to be involved in jasmonic acid production, pollen maturation, and anther dehiscence (Ishiguro et al. 2001). It also negatively regulates disease resistance to the necrotic fungal pathogen *Botrytis cinerea* and avirulent bacteria *Pseudomonas syringae* by promoting cell death and reducing the efficiency of the hypersensitive response, respectively (Camera et al. 2009).

The development of sequence-based computational tools can be of great help in designing effective measures for understanding the molecular behavior of the unknown proteins. Several machine learning and deep learning-based binary predictors have been developed in the last two decades for the classification of target proteins of interest against the other proteins in the genome (Jones 2019). Machine learning techniques are also used in various biological fields including genomics, proteomics, microarrays, systems biology, evolution, and text mining of biological sequences using natural language processing (NLP) (Larranaga et al. 2006). Several parametric and non-parametric machine learning algorithms are currently available and have routinely been used in the classification or prediction of many proteins (Meher et al. 2017). In this context, the development of a machine-learning model for the prediction of the proteins involved in *in-vivo* haploid induction in crops assumes great significance. The model would not only be useful for the identification of patatin-like and non-patatin-like proteins but also support the functional annotation of patatin-like genes in monocots and dicots. Understanding the molecular mechanism of haploid induction in maize would further be improved using molecular approaches. So far, there is no prediction algorithm available for the classification of patatin-like protein in maize or other crops. Therefore, the present study was aimed at machine learning-based classification of patatin-like protein sequences against the other non-patatin-like proteins across crops. The development of a machine learning-based classifier that can predict the patatin-like protein in advance from the other non-patatin-like proteins assumes great significance in gaining prior knowledge before initiating any experiment.

## Materials and methods

### Collection of datasets

**Fig. 1:**
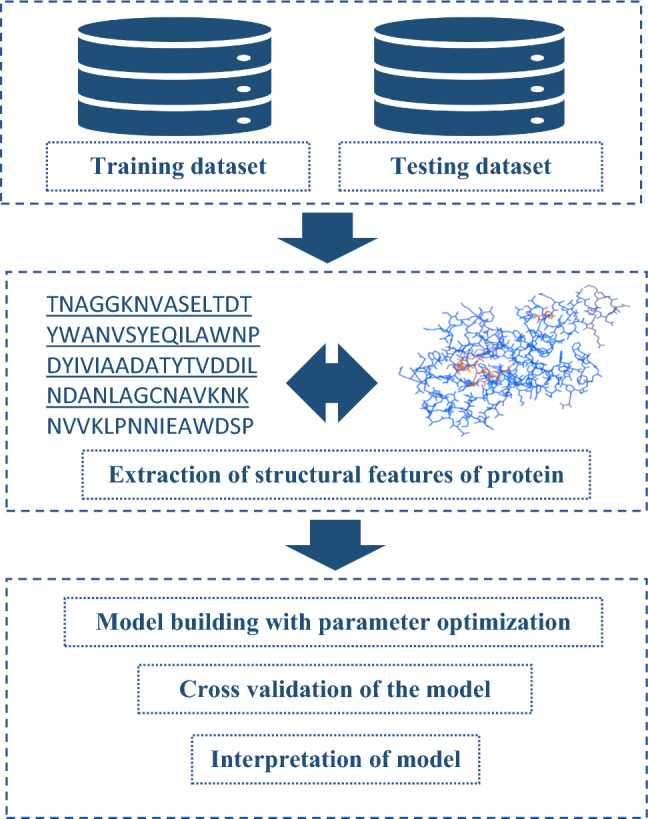
Outline of the model building for patatin-like protein

Two categories of datasets were retrieved from Uniprot (http://www.uniprot.org/) database for the classification of binary data. The first category of protein was termed as patalin-like protein (585 protein sequences), whereas, the second group of protein was considered as non-patatin-like protein (585 protein sequences). Both datasets were processed to remove the sequences with the repeated unit. The positive dataset contains patatin-like proteins from various plant species including both various monocots, dicots, and microbes whereas, the negative dataset was constituted with protein sequences selected randomly (except patalin-like proteins) from the maize protein collections available in the Uniprot database (Supplementary S1). In the non-patatin group, various subspecies of maize such as *Zea mays subsp. huehuetenangensis*, *Zea mays subsp. mays*, *Zea mays subsp. mexicana*, and *Zea mays subsp. parviglumis* had been included (Supplementary S1). A summary of the model development is given the Fig. 1.

### Feature generation

The generation of features from protein sequences plays a key role in classification problems using any machine learning model. Before being used as an input, numeric feature vectors were created from strings of amino acids of each of the protein sequences in supervised learning classifiers. In the present study, five sequence-based features were generated from the amino acid sequences to map them on numeric vector observations. The features include amino acid compositions (AAC), di-peptide composition (DPC), grouped di-peptide composition (GDPC), composition-transition-distribution (CTD), and grouped amino acid composition (GAAC). A summary of the size of the vector space of each of the data sets was presented in Table 1.

**Table 1:** Total number of parameters used for each feature for classification

Feature	Size of the vector space
AAC	20
DPC	400
GDPC	25
CTDC	39
CTDT	39
GAAC	5

*AAC* Amino acid composition; *DPC* Di-peptide Composition; *GDPC* Grouped Di-Peptide Composition; *CTD-C* Composition; *CTD-D* Distribution; *GAAC* Grouped Amino Acid Composition

#### Amino acid composition (AAC)

AAC is the simplest and most widely used structural feature for representing a protein sequence (Bhasin and Raghava 2004). It is the proportions of amino acid residues present in a protein sequence. For a protein sequence with N residues, AAC for the ith amino acid can be computed as AAC (*i*) = fi/*N*, where *i* = 1–20. Therefore, every protein sequence can be transformed into a vector of 20 numeric observations.

#### Di-peptide composition (DPC)

DPC takes the composition as well as ordering effects of amino acid residues in a string of protein sequences (Saravanan and Goutham 2015). DPC can be computed as DPC (*j*) = M_*j*_/(*N* − 1) for any di-peptide M_*j*_, where *j* = 1–400 (20^2^) and *N* indicates the length of the protein sequence. Therefore, each protein sequence can be converted into a 400-dimensional numeric vector using DPC.

#### Grouped di-peptide composition (GDPC)

The GDPC is another variation of the DPC descriptor with 25 parameters that are defined as:$f(r,s)\, = \,\frac{{N_{rs} }}{N - 1},\ \ \ \ \,\,\,\,\,\,r,\,s\,{{ \in }}\,\{ g1,\,g2,\,g3,g4,g5\}$, where *N*_*rs*_ is the number of tripeptides represented by amino acid type groups *r* and *s*, and *N* is the length of a protein.

#### Composition-transition-distribution (CTD)

CTD features denote distribution patterns of amino acids in a peptide sequence (Cai et al. 2003). For computing these features, 13 types of physicochemical properties were previously used (Dubchak et al. 1999). These include hydrophobicity, normalized van-der-Waals volume, polarity, polarizability, charge, secondary structures, and solvent accessibility. The composition (CTDC) feature can be computed as *C*(*r*) = *N*(*r*)/*N*, where *r* belongs to polar, neutral, and hydrophobic amino acids, *N(r)* is the number of amino acid types, *r* is the encoded sequence and *N* is the length of the sequence. The transition (CTDT) feature can then be calculated as T(r,s) = (N(r,s) + N(s,r))/(N− 1) where *r* and s belong to a combination of (i) polar and neutral, (ii) neutral and hydrophobic, and (iii) hydrophobic and polar residues, *N(r,s)* and *N(s,r)* are the numbers of dipeptides encoded as “*rs*” and “*sr*” respectively in the sequence, while *N* is the length of the sequence (Dubchak et al. 1999).

#### Grouped amino acid composition (GAAC)

According to the physicochemical properties (hydrophobicity, molecular size, and charge), 20 amino acid types are further classified into five categories (Lee et al. 2011). The five categories include the aliphatic group (*g1*: GAVLMI), aromatic group (*g2*: FYW), positively charged group (*g3*: KRH), negatively charged group (*g4*: DE) and uncharged group (*g5*: STCPNQ). Here symbolic code represented single letter code of each of the amino acids viz., *G*: Glycine, *A*: Alanine, *L*: Leucine, *M*: Methionine, *F*: Phenylalanine, *W*: Tryptophan, *K*: Lysine, *Q*: Glutamine, *E*: Glutamic acid, *S*: Serine, *P*: Proline, *V*: Valine, *I*: Isoleucine, *C*: Cysteine, *Y*: Tyrosine, *H*: Histidine, *R*: Arginine, *N*: Asparagine, *D*: Aspartic acid, *T*: Threonine). The frequency of each amino acid group is defined as a GAAC descriptor using the following notation:$f(g)\ \ \ \ = \ \ \ \ \frac{N(g)}{N}\ ,\ \ \ \ \ \ \,\ \ \ \ g{{ \in }}\,\{ g1,g2,g3,g4,g5\}$ and $N(g_t ) = \,\sum {N(t)} ,\,\,\,\,\,\,\,\,t{{ \in }}g$, where *N(g)* denotes the number of amino acids in group *g*, *N(t)* is the number of amino acid type *t,* and *N* is the protein length.

### Support vector machine (SVM) classifier

SVM classifier (Vapnik and Chapelle 2000) was used for the classification of patatin-like proteins. SVM is a non-parametric method as it does not make any assumption on the probability distribution of the input dataset. Due to its strong statistical background, SVM can be efficiently employed in various biological studies including computational biology and bioinformatics for classification purposes based on the statistical principle of structural risk minimization (Meher et al. 2017). The ability to handle large and noisy input datasets further makes SVM a more attractive machine-learning tool for classification studies. The performance of SVM highly relies on the type of kernel functions used for tuning the model (Cherkassky and Ma 2004). The role of the kernel function is to map the input dataset on high-dimensional feature space. Initially, 80% of the numeric observations were used with default hyper-parameters of the SVM classifier with four different kernels [radial basis function (RBF), sigmoid, polynomial, and linear]. The kernel(s) for which the highest accuracy was obtained was subsequently used for hypermeter optimization.

### Classification using a balanced dataset

A total of 1170 protein sequences including both patatin-like and non-patatin-like proteins were analyzed in this study. A dataset is called balanced if the number of positive and negative samples are equal, whereas it becomes unbalanced due to the difference between samples belonging to positive and negative groups. In an imbalanced classification problem, the distribution of samples in the training data set is biased or skewed. Machine learning-based classifiers may generate biased results varying from a slight to a severe imbalance. This results in machine learning models that have poor predictive performance, particularly for the minority class. Therefore, binary classification was carried out using a balanced dataset consisting of 585 protein sequences from each group (patatin-like and non-patatin-like) of proteins.

### Evaluation of model performance

Model performance was evaluated through analysis of the confusion matrix, where actual and predicted patatin-like and non-patatin-like proteins were presented as true positive (TP), false positive (FP), false negative (FN), and true negative (TN) categories. Based on actual and predicted observations, several scores were calculated [precision, recall, accuracy score, f1-score, Matthew’s correlation coefficient (MCC)] to evaluate the performance of the predicted model. Equations for the calculation of the different scores were presented in the following-

i. Precision = $$\frac{TP}{TP+FP}$$
ii. Recall = $$\frac{TP}{TP+FN}$$
iii. F1 score = $$2*\frac{precision * recall }{precision + recall}$$
iv. Accuracy = $$\frac{TP+TN}{TP+FN+TN+FP}$$
v. (v) MCC = $$\frac{TP*TN-FP*FN}{\sqrt{\left(TP+FP\right)\left(TP+FN\right)\left(TN+FP\right)\left(TN+FN\right)}}$$

A receiver operating characteristic (ROC) curve was also used as a criterion for evaluating the model performance using different kernels. The ROC curve was plotted using a true positive rate vs. a false positive rate. The area under the curve (AUC) computed using each of the kernels is used for the evaluation of the model performance.

### Evaluation of model performance using cross-validation

Cross-validation is a statistical technique used to evaluate the performance of machine learning algorithms. The performance of the machine learning model was analyzed using tenfold cross-validation. For validation purposes, k-fold and repeated k-fold cross-validation were used to assess the performance of the binary classifier*.* In k-fold cross-validation, the whole data set was divided into k subsets. Now the cross-validation is repeated k times in such a way that one of the k subsets is used as the test/validation set at each time, and the remaining k-1 subsets are assembled together to form the training data set*.* Total effectiveness of the model error estimation averaged over all k trials*. *This reduces bias significantly as most of the data are used for fitting. However, a single run of the k-fold cross-validation may result in a noisy estimate of model performance. Contrarily, repeated k-fold cross-validation provides a measure to improve the estimated performance of a machine learning algorithm as it involves repeated cross-validation procedures multiple times and reporting the mean result across all folds from all runs (Rodriguez et al. 2009). In both cases, tenfold cross-validation was performed using all the kernels of SVM.

### Learning curve analysis for the SVM models

The main goal while developing any machine learning model is to keep errors as minimal as possible. The major sources of error in any machine-learning algorithm are bias and variance (Dietterich and Kong 1995). Therefore, the main goal is to develop a model with low error by keeping both bias and variance at their minimum. However, this is hardly possible to get a model of low bias and low variance. Therefore, there is a trade-off between bias and variance while building a machine learning model. In practice, learning curves usually provide a trade-off between bias and variance based on the performance of training and cross-validation (testing) datasets. The learning curve gives an idea of how well the model is learning from the training dataset. In the present study, the learning curve was plotted for both training and cross-validation scores against the size of training data sets with tenfold cross-validation. Mean training scores and cross-validation scores were presented along with their standard deviation in the learning curve.

### Hypermeter optimisation of SVM model

In SVM, C is the regularization parameter used to control errors in the training data set (Cherkassky and Ma 2004). C parameter usually adds a penalty for each misclassified data point. Due to the small value of C, a decision boundary with a large margin is chosen at the cost of a large number of misclassifications and hence, the penalty for misclassified points is low. SVM tries to minimize the number of misclassified examples while using a large value of C which results in a decision boundary with a smaller margin. The penalty is directly proportional to the distance to the decision boundary and is not the same for all misclassified examples. Gamma is another important parameter that defines how far the effect of a single training example reaches (Meyer and Wien 2015). High gamma value considers only the points close to the plausible hyperplane, whereas low gamma considers points at a greater distance. To find out the optimum combination, GridSearchCV was used on both C (ranging from 10^–3^ to 10^7^ with tenfold intervals) and gamma (ranging from 10^–5^ to 10^3^ with tenfold intervals) parameters in all possible combinations. A stratified k-fold module with tenfold cross-validation was used in this exhaustive search.

### Feature importance of the parameters

To identify the relevant features that contribute the maximum explanation towards output classification, mutual information was used as a selection criterion. Based on ‘information theory’, the mutual information of two random variables quantifies the mutual dependence between the two variables based on their entropy. It estimates the amount of information obtained about one random variable while observing another variable. This can be defined as *I*(X;Y) = *D*_KL_(*P*_(X,Y)_||*P*_X_*P_Y_), where *D*_KL_ denotes Kullback–Leibler divergence, *P*_X_ and *P*_Y_ are the marginal distributions and *P*_(X,Y)_ is the joint distribution of two random variables *X* and *Y*. If two random variables are independent, I(X;Y) become zero since their joint distribution coincides with the product of the marginal distribution. On the other hand, higher values of I(X;Y) indicate greater dependency on the output classes and input features. Using mutual information, most explanatory features were identified in the selected dataset for which the highest accuracy was obtained.

### SVM model comparison with other classification models

Comparisons were made between the newly constructed SVM model with a decision tree, random forest, and logistic regression using the DPC dataset. Based on the actual and predicted observations, a similar set of measures, including accuracy score, precision, recall, f1-score, and MCC, were constructed to compare the model.

### Statistical analysis

All the statistical analysis was carried out in the Anaconda Jupiter Notebook integrated development environment (4.8.3) backed with Python Version 3.7. Microsoft Excel Version 2019 was used for data curation and labeling of the sample. NumPy (v1.18.1) was used to enable numerical computing with Python. An open-source library Pandas (1.0.3) was used for handling the data structure in the analysis. Matplotlib (3.1.3) and Seaborn (0.10.1) were used for creating static and statistical visualizations. SVM classification, hypermeter optimization, and feature importance were performed using the Scikit-learn machine learning library (0.22.1). Scripts for implementing the scoring matrices were added as a Supplementary S2 file.

## Results

### Performance of SMV model using different kernels

Among a total of 1170 sequences including both patatin-like and non-patatin-like proteins, a sample size of 936 protein sequences was used for training the model, and the remaining 234 peptide sequences were included in the testing purpose. Prediction accuracies for the protein were analyzed using different kernels of SVM with default parameters (Table 2). It was observed that prediction accuracies were more precise for the polynomial kernel (95%) followed by RBF (94%), sigmoid (82%), and linear (69%), while DPC was used for the prediction purpose. Precision rates of polynomial, RBF, linear, and sigmoid kernels were 95, 94, 79, and 82%, respectively. Recall values were 95, 94, 69, and 82% for polynomial, RBF, linear, and sigmoid kernel, respectively. Recorded F1-score and MCC were also highest for the polynomial kernel (95 and 90%) as compared to RBF (94 and 88%), linear (66 and 47%), and sigmoid (82 and 65%) kernel in the training data set, respectively. AUC values were 95, 94, 69, and 82% for polynomial, RBF, linear, and sigmoid kernels, respectively. The accuracies, precision, recall, MCC, F1-score, and AUC values are presented in Table 2. Contrary to DPC, the performance of the model was not satisfactory using the GDPC dataset. Model performance with AAC and GAAC datasets was also received poorly using all the kernels of SVM (Table 2). Similarly, the desired label of accuracies for CTDC and CTDT was not obtained using the same hyperparameters (Table 2). Therefore, DPC was identified as the most suited parameter for the classification of patatin-like protein sequences.

**Table 2:** Total number of parameters used for each feature for classification

	RBF	Linear	Sigmoid	Polynomial
AAC				
Accuracy	0.884	0.648	0.572	0.872
Precision	0.884	0.735	0.576	0.872
Recall	0.884	0.648	0.572	0.872
F1-score	0.884	0.612	0.567	0.872
MCC	0.768	0.373	0.147	0.744
AUC	0.88	0.64	0.57	0.87
GAAC				
Accuracy	0.63	0.50	0.50	0.63
Precision	0.65	0.50	0.55	0.67
Recall	0.63	0.50	0.50	0.63
F1-score	0.62	0.49	0.37	0.60
MCC	0.28	0.01	0.04	0.30
AUC	0.63	0.50	0.50	0.63
DPC				
Accuracy	0.94	0.69	0.82	0.95
Precision	0.94	0.79	0.82	0.95
Recall	0.94	0.69	0.82	0.95
F1-score	0.94	0.66	0.82	0.95
MCC	0.88	0.47	0.65	0.90
AUC	0.94	0.69	0.82	0.95
GDPC				
Accuracy	0.71	0.584	0.572	0.744
Precision	0.73	0.647	0.584	0.748
Recall	0.71	0.584	0.572	0.744
F1-score	0.71	0.534	0.556	0.743
MCC	0.44	0.223	0.155	0.492
AUC	0.71	0.58	0.57	0.74
CTDC				
Accuracy	0.79	0.72	0.59	0.82
Precision	0.79	0.72	0.59	0.82
Recall	0.79	0.72	0.59	0.82
F1-score	0.79	0.72	0.58	0.82
MCC	0.58	0.44	0.19	0.64
AUC	0.79	0.72	0.59	0.82
CTDT				
Accuracy	0.82	0.72	0.46	0.84
Precision	0.82	0.73	0.31	0.84
Recall	0.82	0.72	0.46	0.84
F1-score	0.82	0.72	0.32	0.84
MCC	0.64	0.45	− 0.17	0.68
AUC	0.82	0.72	0.46	0.83

*AAC* Amino acid composition; *DPC* Di-peptide Composition; *GDPC* Grouped Di-Peptide Composition; *CTD-C* Composition; *CTD-D* Distribution; *GAAC* Grouped Amino Acid Composition

### Cross-validation of the model performance

**Fig. 2:**
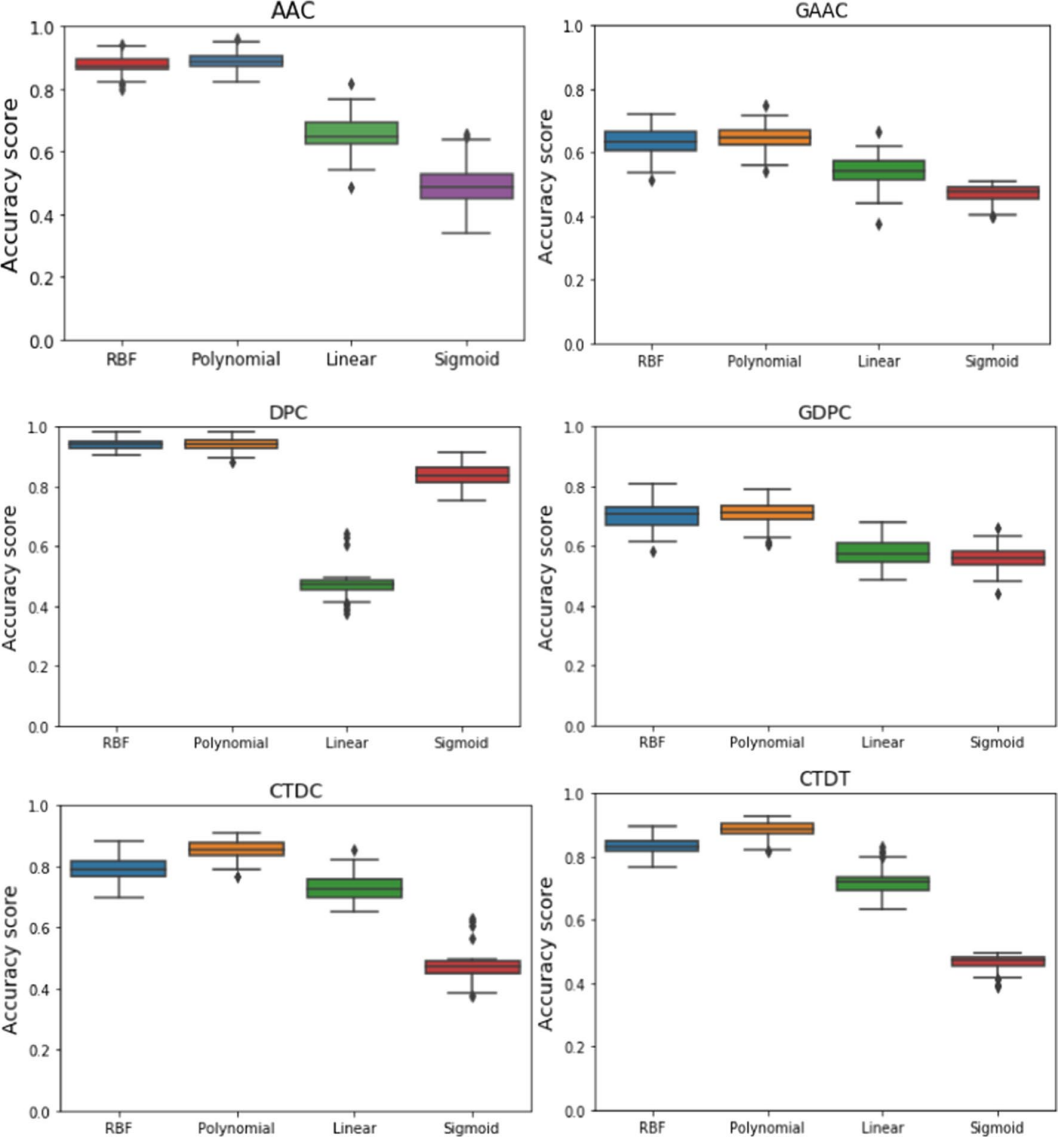
Cross validation results of six different parameters using four different kernels of SVM

The performance of each SVM model with different kernels was analyzed by tenfold cross-validation using stratified k-fold and repeated k-fold cross-validation. Box plots have been presented to visualize the performance of the SVM model with different kernels for all six parameters (Fig. 2). It was observed that model performance was quite satisfactory when DPC was used as the parameter for model training. Performance with RBF and polynomial kernels was at par in all the different datasets (Fig. 2). Using stratified k-fold cross-validation, model performance showed greater than 80% accuracy using ACC, DPC, and CTDT datasets when RBF kernel was used. Model accuracy was more than 80% using the DPC dataset when kernels were used. In the case of the polynomial kernel, model accuracies were greater than 80% using AAC, CTDC, and CTDT datasets, and 92% accuracy was observed for the DPC dataset (Table 3). Similar kind of results were obtained from repeated kFold cross-validation. Using AAC and CTDT datasets, more than 80% accuracy was obtained. whereas 93% accuracy was observed using the DPC dataset. Model accuracy using the DPC dataset was observed to be more than 80% when kernels were used. Using a polynomial kernel, more than 80% accuracy was noticed using AAC, CTDD, and CTDT datasets. In this case, the highest accuracy (94%) was observed for the polynomial kernel using the DPC dataset (Table 3). Therefore, RBF and polynomial kernels were used further for hyper-parameter tuning for patatin-like function prediction.

**Table 3:** Cross validation score using different kernels of SVM

	k-fold cross validation				Repeated k-Fold cross validation			
Kernel	RBF	Linear	Sigmoid	Polynomial	RBF	Linear	Sigmoid	Polynomial
AAC	0.86 ± 0.05	0.63 ± 0.02	0.56 ± 0.06	0.87 ± 0.03	0.87 ± 0.03	0.65 ± 0.05	0.50 ± 0.06	0.88 ± 0.02
GAAC	0.62 ± 0.03	0.53 ± 0.03	0.50 ± 0.01	0.64 ± 0.05	0.63 ± 0.03	0.54 ± 0.04	0.46 ± 0.02	0.64 ± 0.04
DPC	0.91 ± 0.02	0.57 ± 0.10	0.82 ± 0.03	0.92 ± 0.03	0.93 ± 0.01	0.47 ± 0.03	0.83 ± 0.03	0.94 ± 0.02
GDPC	0.68 ± 0.04	0.58 ± 0.04	0.55 ± 0.02	0.70 ± 0.06	0.70 ± 0.04	0.57 ± 0.04	0.56 ± 0.03	0.71 ± 0.03
CTDC	0.76 ± 0.04	0.71 ± 0.07	0.53 ± 0.05	0.84 ± 0.04	0.78 ± 0.03	0.72 ± 0.04	0.46 ± 0.03	0.85 ± 0.03
CTDT	0.82 ± 0.04	0.71 ± 0.04	0.47 ± 0.03	0.85 ± 0.04	0.83 ± 0.02	0.71 ± 0.03	0.46 ± 0.02	0.88 ± 0.02

*AAC* Amino acid composition; *DPC* Di-peptide Composition; *GDPC* Grouped Di-Peptide Composition; *CTD-C* Composition; *CTD-D* Distribution; *GAAC* Grouped Amino Acid Composition; data were presented as mean accuracy ± standard deviation

### Learning curve analysis using different kernels of the SVM

**Fig. 3:**
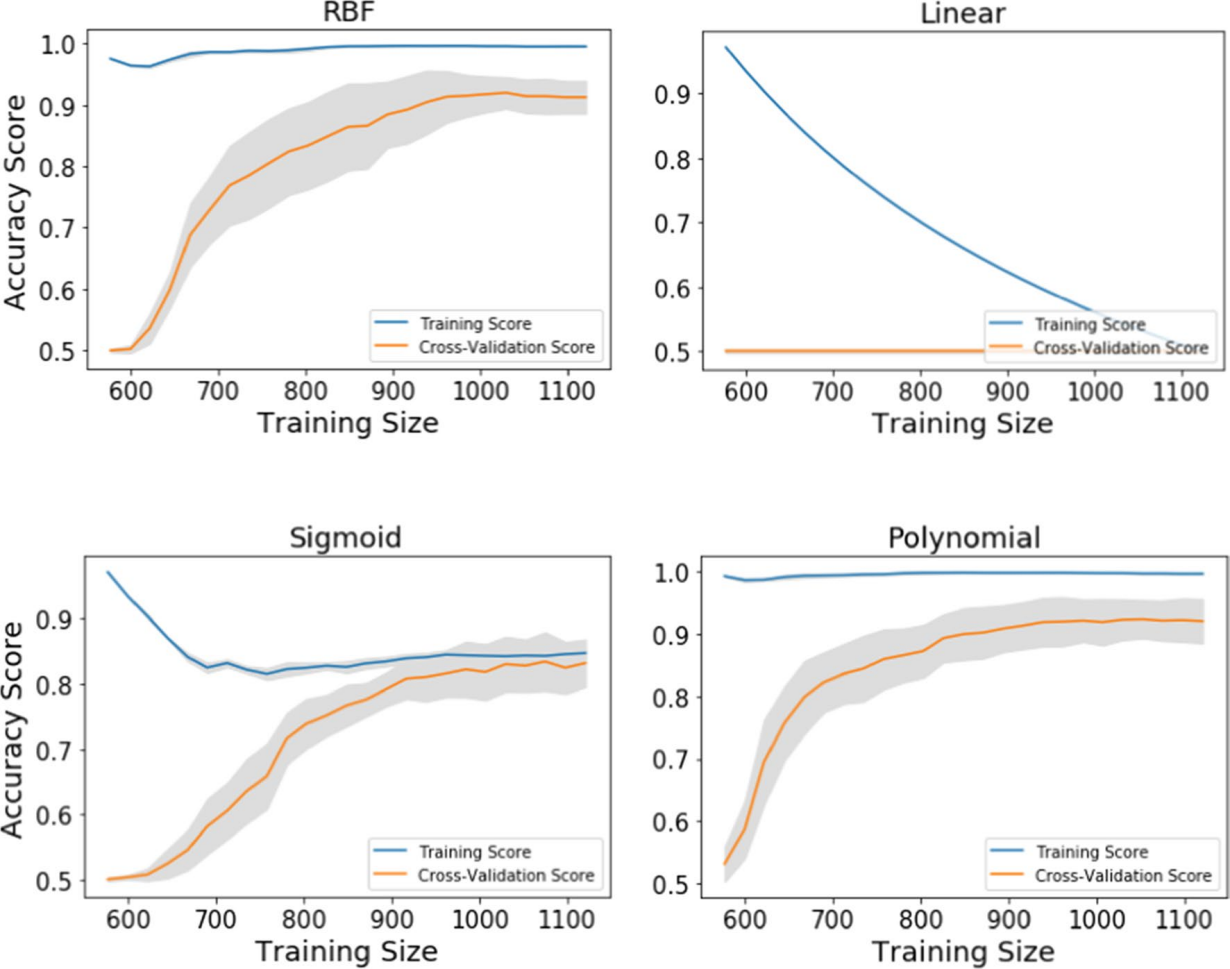
Leaning curve of SVM model with training and cross-validation scores

The learning curve was plotted for both training and cross-validation scores against the size of training data sets with tenfold cross-validation to interpret the performance of the four SVM models with different kernels (Fig. 3). In graphs, the accuracy score of the training set and test set is marked as the training score and cross-validation score; respectively and were presented along with their standard deviation in the learning curve. In the case of the RBF kernel, up to 900 samples the training score was much higher than that of the testing score. However, training and test scores have not yet converged, therefore this model would benefit potentially following the addition of more training data. In the case of the linear kernel, the SVM model failed to train the dataset resulting continuous decline in the training score following increment of the training size. In the sigmoid kernel, there was a huge gap between the training and testing score up to 900 training size and a concomitant decline in the training score of the model. Even though the model converged nearly following the addition of more samples in the training dataset, the accuracy score kept on increasing indicating the potentiality of the model to improve further following the addition of more data in the training dataset. The learning curve of the polynomial model was quite similar to the RBF kernel where up to 900 samples in the training dataset, there was a gap between the training and testing score of the model. In the present study, the performance of RBF and polynomial model was observed more generalized as compared to the SVM model using sigmoid and linear kernels. The model built using RBF and polynomial performed well for the training dataset, however, achieved poor performance on the test dataset indicating a near overcomplicated model with low bias and high variance.

### Hyperparameter optimisation

**Fig. 4:**
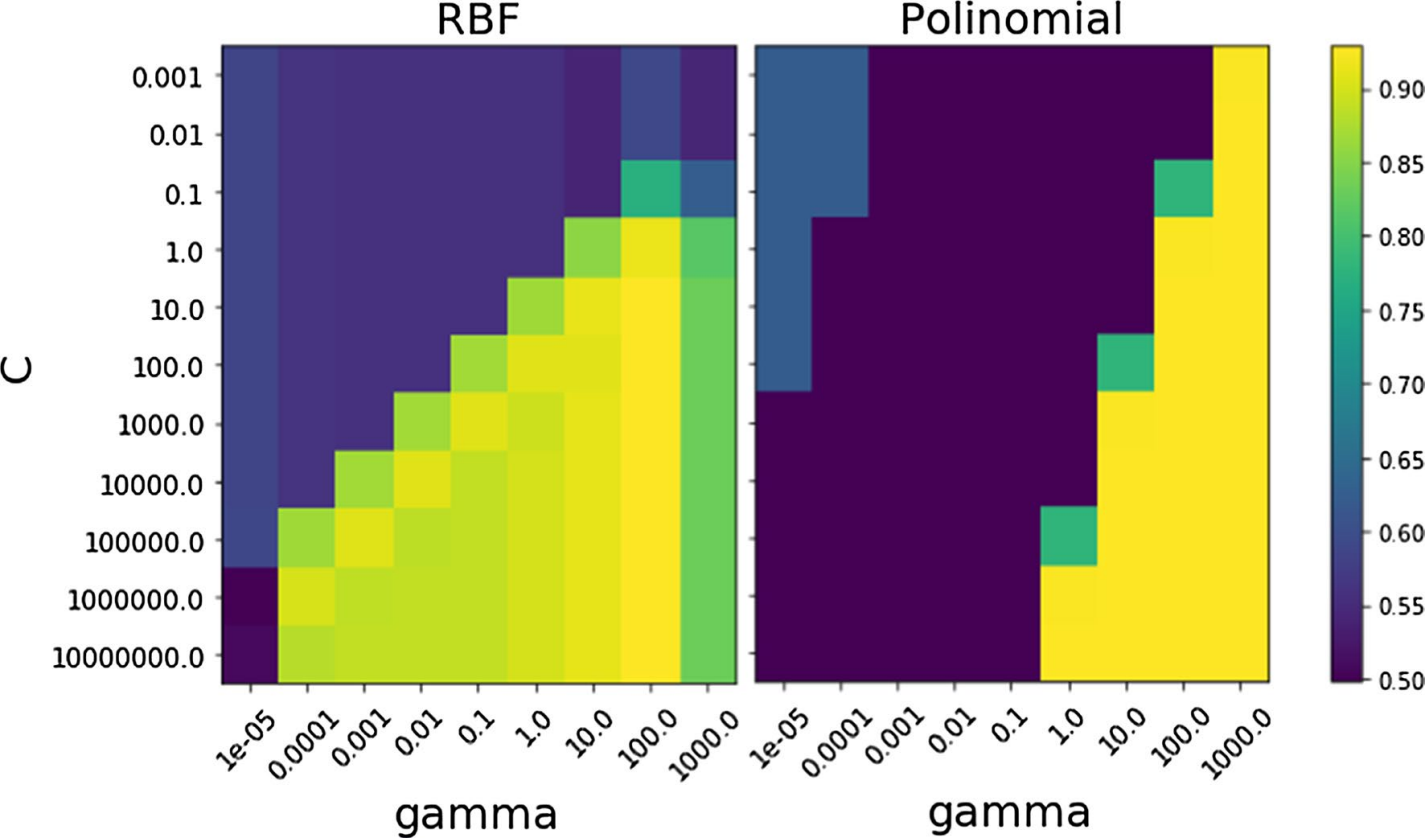
Optimisation of C and gamma parameters of SVM model using RBF and Polynomial kernels; Accuracy scores at all the possible combination (tenfold intervals) of these two parameters were presented using color bar

To further optimize the SVM model, two important parameters, C (regularization parameter) and gamma, were considered at specified intervals in all the possible combinations. The accuracy scores with tenfold cross-validation obtained in each pair of a combination of the C and gamma values were presented using a color bar (Fig. 4). The best RBF classifier (more than 90% accuracy) was detected from the range of 1.0–10^7^ (tenfold intervals) for C and with the gamma value of 10^2^. On the other hand, a wide-range combination of C (10^–1^–10^7^) and gamma (1.0–10^3^) provided more than 90% accuracy using a polynomial kernel. However, the performance of the SVM model using the RBF kernel was far better at a wider range of gamma and *C* values (Fig. 4). Therefore, both C and gamma parameters were important for obtaining a better accuracy score in the prediction of the patatin-like proteins.

### Feature importance of the parameters

**Fig. 5:**
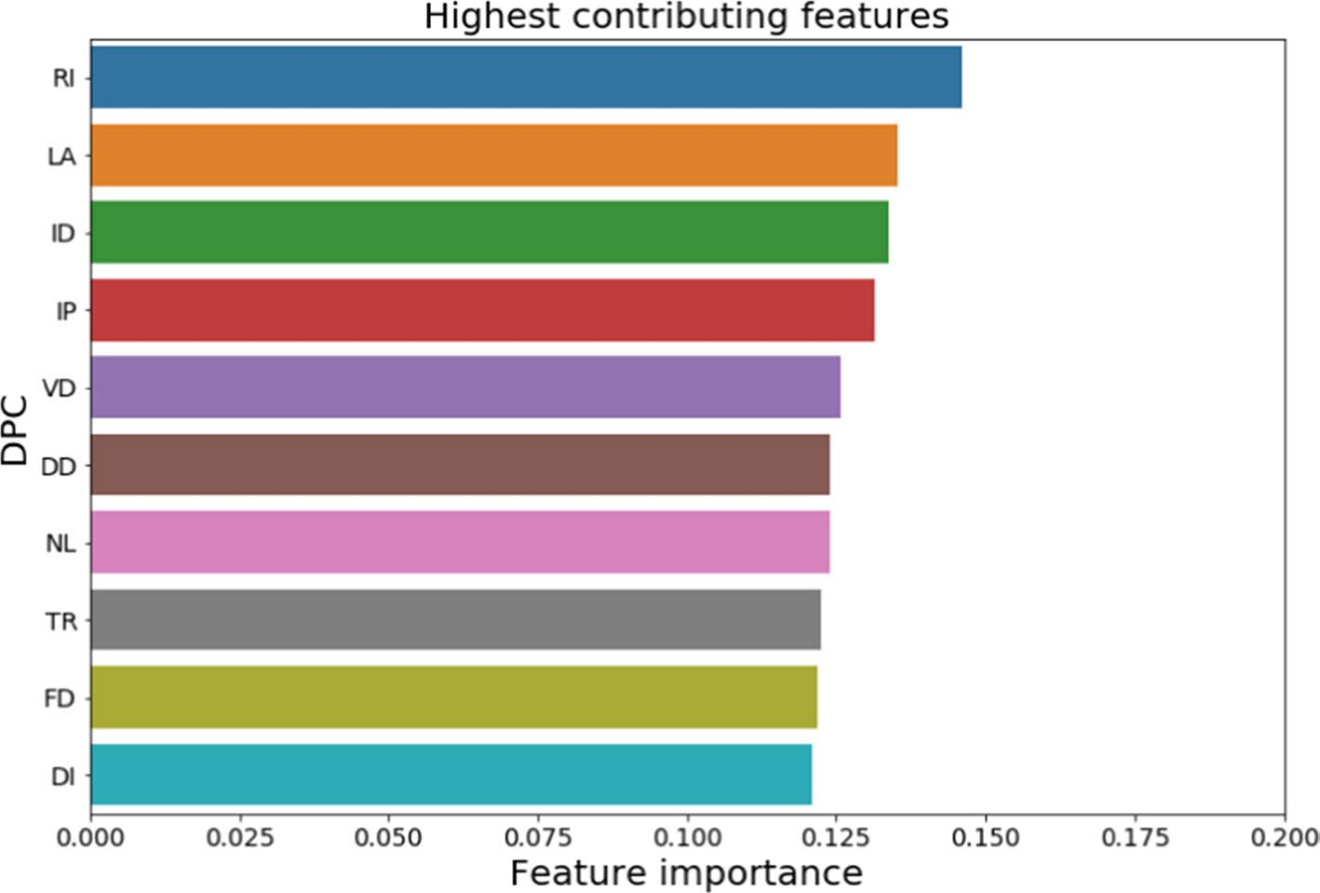
Selection of the features in the DPC dataset; only most important 10 features were shown; dipeptide composition was shown as single letter code of each of the amino acids

To identify the most relevant features that contributed maximum explanation towards output classification, mutual information was used as a selection criterion. The most important dipeptide composition was identified of which the top 10 were visualized (Fig. 5). based on mutual information of two random variables, RI was found to be the most important feature (0.1459) in the DPC dataset followed by LA (0.1353), ID (0.1337), IP (0.1315), VD (0.1258), DD (0.1239), NL (0.1239), TR (0.1224), FD (0.1217) and DI (0.1211). On the other side, the 10 least important features were WC (0.0000), MM (0.0011), FM (0.0069), PC (0.0090), MC (0.0094), WS (0.0097), QM (0.0101), WM (0.0107), CW (0.0111) and YK (0.0145). Dipeptides were presented as single-letter codes of each of the amino acids.

### Performance of other models in protein structure prediction

Using a decision tree, random forest, and logistic regression, 79.60, 91.20, and 82.40% accuracy were obtained using the DPC dataset (Table 4). When compared to the recently created SVM model, the performance of random forest was relatively comparable, while decision tree and logistic regression could not outperform the newly built SVM model. A comparable set of scoring metrics, including precision, recall, f1-score, and MCC, was computed for decision tree, random forest, and logistic regression based on the actual and predicted protein classes (Table 4).

**Table 4:** Comparisons of other classifiers using the DPC dataset

	Decision tree	Random forest	Logistic regression
Accuracy	0.796	0.912	0.824
Precision	0.789	0.948	0.805
Recall	0.808	0.872	0.856
F1-score	0.798	0.908	0.829
MCC	0.592	0.827	0.649

## Discussion

In maize, the *mtl* gene encoding phospholipase contains a patatin-like phospholipase domain that triggers maternal haploid production (Liu et al. 2017). It was found that a 4-bp insertion in the last exon of the *mtl* gene is the underlying factor for the formation of haploid embryos from maternal genotypes (Kelliher et al. 2017). In addition to haploid induction, patatin-like phospholipase group of proteins is also involved in non-specific hydrolysis of the galactolipids (Kobayashi et al. 2007), phospholipids (Camera et al. 2005), besides involved in jasmonic acid production, pollen maturation and anther dehiscence (Ishiguro et al. 2001) and regulating disease resistance (Camera et al. 2009). Here, we developed a machine-learning model for prediction of the both patatin-like and non-patatin-like proteins.

In this present investigation, combinations of AAC, DPC, GDPC, CTDC, CTDT, and GAAC features were used to map the peptide sequences onto numeric feature vectors which were subsequently used as input in SVM for prediction of patatin-like proteins. It is also desirable to know the relationship between the compositional properties of patatin-like proteins and their function concerning biochemical properties relevant to haploid induction and lipid hydrolysis. In this study, DPC was found more predictive as compared to the other five datasets. Huang et al. (2012) also used DPC for the prediction and analysis of protein solubility using a novel scoring card method. Meher et al. (2017) used various compositional (AAC, normalized-AAC, pseudo-AAC), structural (α -helix propensity, β-sheet propensity, turn propensity), and physicochemical (iso-electric point, hydrophobicity, and net-charge) features for prediction of the antimicrobial peptides using SVM classifier. In another study, various features like AAC, DPC, Gap-Pair Composition, pseudo-AAC, CTD, and auto-correlation function were used for the prediction of nitrogen-fixation proteins of diazotrophs, among which CTD was selected as a promising feature for the prediction purpose using SVM classifier with greater than 90% accuracies (Meher et al. 2019).

The present study also suggested that the performance of the SVM model with RFB and polynomial kernels was better. At the same time, DPC features were used for the prediction of the patatin-like protein. Idicula-Thomas et al. (2006) proposed an SVM based learning algorithm to predict protein solubility by evaluating three feature sets. In another study, a large dataset was used for building a two-layered predictor PROSO combining SVM and Naive Bayes classifiers for studying protein solubility (Smialowski et al. 2007). Magnan et al. (2009) used a huge dataset of 17,408 protein sequences and developed a two-stage SVM classifier using SVM and Naive Bayes classifiers. SVM has a distinguishing characteristic that sets it apart from other machine learning techniques: it searches for hyperplanes that linearly segregate positive and negative training data in feature spaces of increasing size. If linear separation is not possible in the supplied feature space, the data are transferred into a higher-dimensional space where linear separation might be achievable (Rodriguez-Perez and Bajorath 2022). The main goal while developing any machine learning model is to keep errors as minimal as possible (Dietterich and Kong 1995). In the present study, the training error of the model using RBF and polynomial kernel was very less indicating the presence of low bias. However, the difference between the training and testing accuracy of the model exists even after the training increases indicating the overfitting of the model due to high variance. Increased regularization or selection of the features are techniques to reduce the complexity of the model. Initially, zero training error occurs because the model fits into a single data point and hence, the fitted line lies exactly on the data point. However, when the model is applied to unseen validation data, it results in a high validation error. As the training size increases, the fitted model minimizes the error over all data points and therefore, does not fit all data perfectly. Hence, eventually, the training error increases, and the validation error decreases as the size of training instances increases. When the curves become plateau after obtaining a certain optimal training data size, increasing size no longer increases the efficiency of the training process. The low error of the training curve gives information about low bias and vice versa. On the contrary, the gap between the error of training and validation curves provides information about the variance. A narrower gap between the training and testing error indicates the presence of low variance and vice versa. A good model generally has low bias and low variance which is eventually very difficult to obtain practically. An oversimplified model, on the other hand, generally contains high bias and low variance, as it does not capture information from data and produces poor prediction. In addition, low bias and high variance lead to an overcomplicated model as it performs well for the training dataset but poor for test dataset due to capturing the random noise present in training data. In SVM, regularization parameters *C* and gamma are the two important parameters to control error in the training data set (Cherkassky and Ma 2004). Due to the small value of *C*, a decision boundary with a large margin is selected at the expense of the large number of misclassifications leading to a low penalty for misclassified points (Duan et al. 2003; Wainer and Cawley 2017). The gamma parameter defines how far the effect of a single training example reaches (Meyer and Wien 2015). High gamma value considers only the points close to the plausible hyperplane, whereas low gamma considers points at a greater distance (Keerthi 2002; Al-Mejibli et al. 2020). Feature in the training dataset in another is also an important parameter that determines the performance of the SVM model. In this study, DPC was selected and was in converted into a 400-dimensional numeric vector. Apart from model performance with a particular dataset, it was also a wonder to know which features in the DPC dataset are the most important in determining the forecast. In this context, feature selection is an important technique to identify the relevant features (di-peptide) that contribute maximum explanation towards output classification. In the present study, using mutual information, the 10 most important dipeptide residues were identified contributing the highest towards predicting the output classes (patatin-like and non-patatin-like). Mutual information is a non-negative value between two random variables measuring dependency between the variables. Higher values depict higher dependency, whereas it becomes zero if two random variables are independent (Kraskov et al. 2004; Ross 2014). The performance of decision tree, random forest, and logistic regression classifiers were also compared with that of SVM. Though the performance of SVM was found *at par* with that of random forest using DPC dataset, it was significantly higher than that of decision tree and logistic regression classifiers. Since a balanced dataset was used for classification purposes, both SVM and random forest performance were similar in the prediction of the protein sequence. However, unbalanced datasets may sometimes lead to varying results using SVM and random forest models (Meher et al. 2016).

The quantity and caliber of the information that is readily available affects the predictability of bioinformatics approaches, which routinely rely on the knowledge contained in biological sequences (Dutta et al. 2023). Additionally, protein structure predictions were considerably more accurate as a result of the expansion of the knowledge included in the Protein Data Bank (Bernstein et al. 1977; Berman et al. 2000) as well as the utilization of evolutionary data drawn from protein sequence databases and assessed with multiple sequence alignments (Cuff and Barton 1999; Simossis and Heringa 2004). The SVM algorithm does not work well with huge data sets or when the target classes are overlapping, which adds more noise to the data set. The SVM will perform poorly when there are more training data samples than features for each data point. There are currently a number of techniques for predicting the local backbone conformation of protein residues that are useful tools in molecular biology (Frishman and Argos 1995; Rost et al. 2004). Furthermore, it is clear that more experimental data will enable better forecasts to be made. Without knowledge of sequence information, no predictions are feasible using any computational tools. On the other hand, even if an endless amount of experimental data were to become available, it would be hardly possible to forecast the perfection of prediction methods. A different angle can be taken to view this uncertainty while comparing the accuracy of two or more prediction systems (Carugo 2007). Furthermore, they should be compared on the same data sets, which is not always possible due to the dynamic nature of biological databases, where new entries may replace old entries in a database. As a result, both the data and the learning algorithms are crucial to the long-term success of SVM-associated applications. Machine learning models may lose their usefulness or perform less accurately if the training data are unsuitable for learning, such as non-representative, low-quality, irrelevant features, or insufficient in quantity (Sarker 2021). For a machine learning-based solution and finally developing intelligent apps, it is crucial to handle the data and various learning algorithms efficiently. To bridge the information gap and to get a deeper understanding of the protein of study in both material and informational dimensions, experimental validation of bioinformatically produced hypotheses and in silico predictions should be triangulated with *in-vitro* and *in-vivo* methodologies (Laub et al. 2023).

So far, no online tool is available to detect a protein having patatin-like activity, thereby posing serious limitations in undertaking in-depth analysis of many such proteins in crops especially those involved in *in-vivo* haploid induction. Here, we also proposed the first machine learning model to computationally identify the two categories of proteins (patatin-like and non-patatin-like). Machine learning algorithms are effective enough to handle sizable datasets with high levels of noise, dimensionality, and/or incompleteness and make few assumptions about the probability distributions and generation processes used to create the data (Mahood et al. 2020). Although in practice the comparison between machine learning and statistics is rather hazy, the main focus of machine learning methods is prediction, which differs from the inferential focus of conventional statistical approaches (Bzdok et al. 2018). SVMs have been demonstrated to be effective in multi-class problems as well as binary classification issues (Mathur and Foody 2008). The developed model is expected to supplement the transcriptional profiling and comparative genomics studies for the identification and functional annotation of genes related to *in-vivo* maternal haploid induction. The model will not only be useful for the identification of patatin-like and non-patatin-like proteins but also support the functional annotation of patatin-like genes on the genome of many monocot species. The developed model not only represents the future direction for developing other computational methods but is also important for most of the experimental scientists working in the field of *in-vivo* haploid induction. This is the first report of machine learning of the identification of proteins with patatin-like activity in crops. The developed model can be used for the development of an online server portal to detect the unknown protein with phospholipase patatin-like activity. The SVM model with RBF and polynomial kernel with specified parameters can be easily used by the researchers for proteome-wide identification of patatin-like proteins without going into details of the statistical methods adopted in developing the approach.

## Supplementary Information

Below is the link to the electronic supplementary material.

Supplementary file1 (XLSX 61 KB)

Supplementary file2 (IPYNB 9 KB)

## Declarations

### Data availability

All the data sets supporting the conclusion of this article are included within the article, and its supporting information files are provided as accompanying supplementary materials S1.

## References

[CR1] Al-Mejibli IS, Alwan JK, Abd Dhafar H (2020) The effect of gamma value on support vector machine performance with different kernels. Int J Electr Comput Eng. 10:5497. 10.11591/ijece.v10i5.pp5497-5506

[CR2] Berman HM, Westbrook J, Feng Z, Gilliland G, Bhat TN, Weissig H, Shindyalov IN, Bourne PE (2000) The protein data bank. Nucleic Acids Res 28:235–242. 10.1093/nar/28.1.235 10592235 10.1093/nar/28.1.235 PMC102472

[CR3] Bernstein FC, Koetzle TF, Williams GJ, Meyer EF Jr, Brice MD, Rodgers JR, Kennard O, Shimanouchi T, Tasumi M (1977) The protein data bank: a computer-based archival file for macromolecular structures. J Mol Biol 112:535–542. 10.1016/S0022-2836(77)80200-3 875032 10.1016/s0022-2836(77)80200-3

[CR4] Bhasin M, Raghava GP (2004) Classification of nuclear receptors based on amino acid composition and dipeptide composition. J Biol Chem 279:23262–23266. 10.1074/jbc.M401932200 15039428 10.1074/jbc.M401932200

[CR5] Bzdok D, Altman N, Krzywinski M (2018) Statistics versus machine learning. Nat Methods 15:233–234. 10.1038/nmeth.4642 30100822 10.1038/nmeth.4642 PMC6082636

[CR6] Cai CZ, Han LY, Ji ZL, Chen X, Chen YZ (2003) SVM-Prot: web-based support vector machine software for functional classification of a protein from its primary sequence. Nucleic Acids Res 31:3692–3697. 10.1093/nar/gkg600 12824396 10.1093/nar/gkg600 PMC169006

[CR7] Camera SL, Balague C, Gobel C, Geoffroy P, Legrand M, Feussner I, Roby D, Heitz T (2009) The Arabidopsis patatin-like protein 2 (PLP2) plays an essential role in cell death execution and differentially affects biosynthesis of oxylipins and resistance to pathogens. Mol Plant Microbe Interact 22:469–481. 10.1094/MPMI-22-4-0469 19271961 10.1094/MPMI-22-4-0469

[CR8] Carugo O (2007) Recent progress in measuring structural similarity between proteins. Curr Protein Pept Sci 8:219–241. 10.2174/138920307780831839 17584118 10.2174/138920307780831839

[CR9] Chaikam V, Gowda M, Nair SK, Melchinger AE, Boddupalli PM (2019) Genome-wide association study to identify genomic regions influencing spontaneous fertility in maize haploids. Euphytica 215:138. 10.1007/s10681-019-2459-5 31402796 10.1007/s10681-019-2459-5 PMC6647887

[CR10] Chaikam V, Nair SK, Martinez L, Lopez LA, Utz HF, Melchinger AE, Boddupalli PM (2018) Marker-assisted breeding of improved maternal haploid inducers in maize for the tropical/subtropical regions. Front Plant Sci 9:1527. 10.3389/fpls.2018.01527 30405665 10.3389/fpls.2018.01527 PMC6201356

[CR11] Cherkassky V, Ma Y (2004) Practical selection of SVM parameters and noise estimation for SVM regression. Neural Netw 17:113–126. 10.1016/S0893-6080(03)00169-2 14690712 10.1016/S0893-6080(03)00169-2

[CR12] Coe EH Jr (1959) A line of maize with high haploid frequency. Am Nat 93:381–382. 10.1086/282098

[CR13] Cuff JA, Barton GJ (1999) Evaluation and improvement of multiple sequence methods for protein secondary structure prediction. Proteins 34:508–519. 10.1002/(SICI)1097-0134(19990301)34:4%3c508::AID-PROT10%3e3.0.CO;2-4 10081963 10.1002/(sici)1097-0134(19990301)34:4<508::aid-prot10>3.0.co;2-4

[CR14] Dietterich TG, Kong EB (1995) Machine learning bias, statistical bias, and statistical variance of decision tree algorithms. Technical report, Department of Computer Science, Oregon State University

[CR15] Dong X, Xu X, Miao J, Li L, Zhang D, Mi X, Liu C, Tian X, Melchinger AE, Chen S (2013) Fine mapping of qhir1 influencing in vivo haploid induction in maize. Theor Appl Genet 126:1713–1720. 10.1007/s00122-013-2086-9 23539086 10.1007/s00122-013-2086-9

[CR16] Duan K, Keerthi SS, Poo AN (2003) Evaluation of simple performance measures for tuning SVM hyperparameters. Neurocomputing 51:41–59. 10.1016/S0925-2312(02)00601-X

[CR17] Dubchak I, Muchnik I, Mayor C, Dralyuk I, Kim SH (1999) Recognition of a protein fold in the context of the SCOP classification. Proteins 35:401–407. 10.1002/(SICI)1097-0134(19990601)35:4<401::AID-PROT3>3.0.CO;2-K 10382667

[CR18] Dutta S, Muthusamy V, Zunjare RU, Hossain F (2022) Accelerated generation of elite inbreds in maize using doubled haploid technology. Plant Breeding-New Perspectives, London

[CR19] Dutta S, Zunjare RU, Muthusamy V, Hossain F (2023) Prediction of CENH3 protein in maize using machine learning techniques. Pharma Innovation J 12:01–06. 10.22271/tpi.2023.v12.i7Sa.21185

[CR20] Frishman D, Argos P (1995) Knowledge-based protein secondary structure assignment. Proteins 23:566–579. 10.1002/prot.340230412 8749853 10.1002/prot.340230412

[CR21] Gain N, Chhabra R, Chandra S, Zunjare RU, Dutta S, Chand G, Sarika K, Devi EL, Kumar A, Madhavan J, Muthusamy V (2022) Variation in anthocyanin pigmentation by *R1-navajo* gene, development and validation of breeder-friendly markers specific to *C1-Inhibitor* locus for *in-vivo* haploid production in maize. Mol Biol Rep 50:2221–2229. 10.1007/s11033-022-08214-2 36564657 10.1007/s11033-022-08214-2

[CR22] Gilles LM, Khaled A, Laffaire JB, Chaignon S, Gendrot G, Laplaige J, Berges H, Beydon G, Bayle V, Barret P, Comadran J (2017) Loss of pollen-specific phospholipase NOT LIKE DAD triggers gynogenesis in maize. EMBO J 36:707–717 28228439 10.15252/embj.201796603 PMC5350562

[CR23] Huang HL, Charoenkwan P, Kao TF, Lee HC, Chang FL, Huang WL, Ho SJ, Shu LS, Chen WL, Ho SY (2012) Prediction and analysis of protein solubility using a novel scoring card method with dipeptide composition. BMC Bioinform 13:1–14. 10.1186/1471-2105-13-S17-S3 10.1186/1471-2105-13-S17-S3 PMC3521471 23282103

[CR24] Idicula-Thomas S, Kulkarni AJ, Kulkarni BD, Jayaraman VK, Balaji PV (2006) A support vector machine-based method for predicting the propensity of a protein to be soluble or to form inclusion body on overexpression in *Escherichia coli*. Bioinformatics 22:278–284. 10.1093/bioinformatics/bti810 16332713 10.1093/bioinformatics/bti810

[CR25] Ishiguro S, Kawai-Oda A, Ueda J, Nishida I, Okada K (2001) The defective in anther DEHISCENCE1 gene encodes a novel phospholipase A1 catalyzing the initial step of jasmonic acid biosynthesis, which synchronizes pollen maturation, anther dehiscence, and flower opening in Arabidopsis. Plant Cell 13:2191–2209. 10.1105/tpc.010192 11595796 10.1105/tpc.010192 PMC139153

[CR26] Jones DT (2019) Setting the standards for machine learning in biology. Nat Rev Mol Cell Biol 20:659–660. 10.1038/s41580-019-0176-5 31548714 10.1038/s41580-019-0176-5

[CR27] Keerthi SS (2002) Efficient tuning of SVM hyperparameters using radius/margin bound and iterative algorithms. IEEE Trans Neural Netw 13:1225–1229. 10.1109/TNN.2002.1031955 18244520 10.1109/TNN.2002.1031955

[CR28] Kelliher T, Starr D, Richbourg L, Chintamanani S, Delzer B, Nuccio ML, Green J, Chen Z, McCuiston J, Wang W, Liebler T (2017) MATRILINEAL, a sperm-specific phospholipase, triggers maize haploid induction. Nature 542:105–109. 10.1038/nature20827 28114299 10.1038/nature20827

[CR29] Kobayashi K, Kondo M, Fukuda H, Nishimura M, Ohta H (2007) Galactolipid synthesis in chloroplast inner envelope is essential for proper thylakoid biogenesis, photosynthesis, and embryogenesis. Proc Natl Acad Sci 104:17216–17221. 10.1073/pnas.0704680104 17940034 10.1073/pnas.0704680104 PMC2040463

[CR30] Kraskov A, Stogbauer H, Grassberger P (2004) Estimating mutual information. Phys Rev 69:066138. 10.1103/PhysRevE.69.066138 10.1103/PhysRevE.69.066138 15244698

[CR31] La Camera S, Geoffroy P, Samaha H, Ndiaye A, Rahim G, Legrand M, Heitz T (2005) A pathogen-inducible patatin-like lipid acyl hydrolase facilitates fungal and bacterial host colonization in Arabidopsis. Plant J 44:810–825. 10.1111/j.1365-313X.2005.02578.x 16297072 10.1111/j.1365-313X.2005.02578.x

[CR32] Larranaga P, Calvo B, Santana R, Bielza C, Galdiano J, Inza I, Lozano JA, Armananzas R, Santafe G, Perez A, Robles V (2006) Machine learning in bioinformatics. Brief Bioinform 7:86–112. 10.1093/bib/bbk007 16761367 10.1093/bib/bbk007

[CR33] Laub V, Devraj K, Elias L, Schulte D (2023) Bioinformatics for wet-lab scientists: practical application in sequencing analysis. BMC Genomics 24:382. 10.1186/s12864-023-09454-7 37420172 10.1186/s12864-023-09454-7 PMC10326960

[CR34] Lee TY, Lin ZQ, Hsieh SJ, Bretana NA, Lu CT (2011) Exploiting maximal dependence decomposition to identify conserved motifs from a group of aligned signal sequences. Bioinformatics 27:1780–1787. 10.1093/bioinformatics/btr291 21551145 10.1093/bioinformatics/btr291

[CR35] Liu C, Li X, Meng D, Zhong Y, Chen C, Dong X, Xu X, Chen B, Li W, Li L, Tian X (2017) A 4-bp insertion at ZmPLA1 encoding a putative phospholipase A generates haploid induction in maize. Mol Plant 10:520–522. 10.1016/j.molp.2017.01.011 28179149 10.1016/j.molp.2017.01.011

[CR36] Magnan CN, Randall A, Baldi P (2009) SOLpro: accurate sequence-based prediction of protein solubility. Bioinformatics 25:2200–2207. 10.1093/bioinformatics/btp386 19549632 10.1093/bioinformatics/btp386

[CR37] Mahood EH, Kruse LH, Moghe GD (2020) Machine learning: a powerful tool for gene function prediction in plants. Appl Plant Sci 8:e11376. 10.1002/aps3.11376 32765975 10.1002/aps3.11376 PMC7394712

[CR38] Mathur A, Foody GM (2008) Multiclass and binary SVM classification: Implications for training and classification users. IEEE Geosci Remote Sens Lett 5:241–245. 10.1109/LGRS.2008.915597

[CR39] Meher PK, Sahu TK, Mohanty J, Gahoi S, Purru S, Grover M, Rao AR (2019) nifPred: proteome-wide identification and categorization of nitrogen-fixation proteins of diaztrophs based on composition-transition-distribution features using support vector machine. Front Microbio 9:1100. 10.3389/fmicb.2018.01100 10.3389/fmicb.2018.01100 PMC5986947 29896173

[CR40] Meher PK, Sahu TK, Rao AR (2016) Prediction of donor splice sites using random forest with a new sequence encoding approach. BioData Mining 9:1–25. 10.1186/s13040-016-0086-4 26807151 10.1186/s13040-016-0086-4 PMC4724119

[CR41] Meher PK, Sahu TK, Saini V, Rao AR (2017) Predicting antimicrobial peptides with improved accuracy by incorporating the compositional, physico-chemical and structural features into Chou’s general PseAAC. Sci Rep 7:1–12. 10.1038/srep42362 28205576 10.1038/srep42362 PMC5304217

[CR42] Meyer D, Wien FT (2015) Support vector machines. The Interface to libsvm in package e1071, 28: 20

[CR43] Prasanna BM, Chaikam V, Mahuku G (2012) Doubled haploid technology in maize breeding: theory and practice. CIMMYT, Mexico

[CR44] Rodriguez JD, Perez A, Lozano JA (2009) Sensitivity analysis of k-fold cross validation in prediction error estimation. IEEE Trans Pattern Anal Mach Intell 32:569–575. 10.1109/TPAMI.2009.187 10.1109/TPAMI.2009.187 20075479

[CR45] Rodriguez-Perez R, Bajorath J (2022) Evolution of support vector machine and regression modeling in chemoinformatics and drug discovery. J Comput Aided Mol Des 36:355–362. 10.1007/s10822-022-00442-9 35304657 10.1007/s10822-022-00442-9 PMC9325859

[CR46] Ross BC (2014) Mutual information between discrete and continuous data sets. PLoS ONE 9:e87357. 10.1371/journal.pone.0087357 24586270 10.1371/journal.pone.0087357 PMC3929353

[CR47] Rost B, Yachdav G, Liu J (2004) The predict protein server. Nucleic Acids Res 32:W321-326. 10.1093/nar/gkh377 15215403 10.1093/nar/gkh377 PMC441515

[CR48] Saravanan V, Gautham N (2015) Harnessing computational biology for exact linear B-cell epitope prediction a novel amino acid composition-based feature descriptor. OMICS 19:648–658. 10.1089/omi.2015.0095 26406767 10.1089/omi.2015.0095

[CR49] Sarker IH (2021) Machine learning: algorithms, real-world applications and research directions. Sn Comput Sci 2:160–181. 10.1007/s42979-021-00592-x 33778771 10.1007/s42979-021-00592-x PMC7983091

[CR50] Simossis VA, Heringa J (2004) Integrating protein secondary structure prediction and multiple sequence alignment. Curr Prot Pept Sci 5:249–266. 10.2174/1389203043379675 10.2174/1389203043379675 15320732

[CR51] Smialowski P, Martin-Galiano AJ, Mikolajka A, Girschick T, Holak TA, Frishman D (2007) Protein solubility: sequence based prediction and experimental verification. Bioinformatics 23:2536–2542. 10.1093/bioinformatics/btl623 17150993 10.1093/bioinformatics/btl623

[CR52] Vapnik V, Chapelle O (2000) Bounds on error expectation for support vector machines. Neural Comput 12:2013–2036. 10.1162/089976600300015042 10976137 10.1162/089976600300015042

[CR53] Wainer J, Cawley G (2017) Empirical evaluation of resampling procedures for optimising SVM hyperparameters. J Mach Learn Res 18:1–35

